# Antibiotic susceptibility patterns of *Salmonella* isolates from clinical, food, and environmental sources in Addis Ababa and surrounding towns, Ethiopia

**DOI:** 10.1128/spectrum.00970-25

**Published:** 2025-07-11

**Authors:** Abebe M. Aga, Demise Mulugeta, Dassalegn Muleta, Adugna Abdi Woldesemayat, Abera Motuma, Musin Kelel, Bilise Wakitole, Shambel Tadesse, Zinash Teferi, Fanos Tadesse Woldemariyam, Serkadis Oljira, Henok Ferede, Natnael Berihun, Samson Girma, Dereje Nigussie, Mesfin Tafesse Gemeda

**Affiliations:** 1 Vaccines, Diagnostics and Medical Device R&D, Armauer Hansen Research Institute 70605, Addis Ababa, Ethiopia; 2 Department of Biotechnology, Biotechnology and Bioprocess Centre of Excellence, Addis Ababa Science and Technology University 446348, Addis Ababa, Ethiopia; 3 Ethiopian Public Health Institute 128164, Addis Ababa, Ethiopia; Montefiore Medical Center and Albert Einstein College of Medicine, Bronx, New York, USA

**Keywords:** clinical isolates, disc diffusion, environmental samples, multidrug resistance, public health, *Salmonella *spp.

## Abstract

The increasing prevalence of antimicrobial resistance in *Salmonella* poses significant challenges to public health globally, particularly in low-resource settings such as Ethiopia. This study aimed to assess the antimicrobial resistance profiles of *Salmonella* isolates from human, food, and environmental sources in Addis Ababa and surrounding towns, with a focus on multidrug resistance (MDR) patterns. A total of 552 samples, consisting of 386 stool samples from patients with diarrhea and 166 food (meat) and environmental samples (such as waste water, cutting board, knife, and chicken dropping), were collected from 12 health facilities and two slaughterhouses. *Salmonella* was isolated and screened through selective culture methods and biochemical tests. Antimicrobial sensitivity tests were done by the Kirby-Bauer disk diffusion method. Among the 61 *Salmonella* isolates tested, the highest resistance was observed against tetracycline (63.9%), followed by ampicillin (55.7%). Resistance to trimethoprim-sulfamethoxazole and chloramphenicol was noted in 24.6% and 19.7% of isolates, respectively. Azithromycin resistance was also sensible at 27.9%. In contrast, ciprofloxacin showed relatively low resistance at 8.2%. Multidrug resistance defined as resistance to three or more antimicrobial classes was detected in 37.7% of isolates. Notably, clinical isolates exhibited significantly higher resistance levels compared to food and environmental sources, with 40% of clinical isolates demonstrating MDR patterns. Gentamicin (3.3%), ceftriaxone (6.6%), and amoxicillin-clavulanic acid (9.8%) remained largely effective, showing low resistance rates. This is one of the few comprehensive studies from Ethiopia examining *Salmonella* resistance across clinical, food, and environmental samples. High antimicrobial resistance, especially to ampicillin and tetracycline, highlights the urgent need for improved surveillance, rational antibiotic use, and infection control. The presence of multidrug-resistant strains stresses the importance of a coordinated national strategy. Future research should focus on developing simple, effective, and point-of-care applicable rapid diagnostic test kits to enable early detection and targeted treatment of multidrug-resistant *Salmonella* in resource-limited settings.

**IMPORTANCE:**

Antibiotic-resistant *Salmonella* is a growing public health threat, particularly in low-resource settings like Ethiopia, where delayed detection and limited treatment options worsen disease outcomes. This study provides a comprehensive analysis of *Salmonella* isolates from clinical, food, and environmental samples in Addis Ababa and nearby towns, offering current data on antimicrobial resistance patterns. By using updated laboratory standards and sampling diverse sources, the findings highlight the urgent need for improved food safety practices, sanitation, and antimicrobial stewardship.

## INTRODUCTION

*Salmonella*, a gram-negative, facultative anaerobic bacterium, is one of the most prevalent causes of foodborne illnesses worldwide, contributing significantly to morbidity and mortality. The pathogen is associated with a wide range of infections, from self-limiting gastroenteritis to invasive bloodstream infections, particularly in immunocompromised individuals (1). The rising prevalence of antibiotic-resistant *Salmonella* strains has emerged as a critical global health challenge, complicating treatment regimens and increasing the burden of disease (2). Globally, non-typhoidal *Salmonella* (NTS) is responsible for an estimated 93.8 million cases of gastroenteritis and 155,000 deaths annually (3). Antibiotic resistance in *Salmonella* is rising due to the overuse and misuse of antibiotics in human medicine and agriculture. A systematic review highlighted multidrug resistance (MDR) in 37% of global NTS isolates, with high resistance rates reported for first-line antibiotics such as ampicillin (AMP), tetracyclines (TET), and sulfonamides (4).

*Salmonella* drug resistance presents a serious global health challenge, affecting both developing and developed countries by leading to treatment failures, longer illness duration, and increased healthcare costs. In the USA, the National Antimicrobial Resistance Monitoring System reported a significant increase in *Salmonella* isolates resistant to ciprofloxacin (CIP), a key drug for treating severe infections (5). In Europe, MDR *Salmonella* outbreaks, such as the 2022 outbreak linked to chocolate products in Belgium, underscore the persistent threat posed by resistant strains even in regions with advanced surveillance and food safety systems (6).

Africa bears a disproportionate burden of antibiotic-resistant *Salmonella* infections, fueled by inadequate healthcare infrastructure, limited detection capacity, and widespread antibiotic misuse (7, 8). Non-typhoidal *Salmonella* infections are particularly problematic in sub-Saharan Africa, where they are a leading cause of invasive bloodstream infections, with case fatality rates ranging from 20% to 25% (9). A meta-analysis of *Salmonella* isolates in Africa revealed alarming resistance patterns: 77% of isolates were resistant to tetracycline, 68% to ampicillin, and 49% to chloramphenicol (CHL) (2). These findings highlight the urgent need for comprehensive surveillance systems and antimicrobial stewardship programs across the continent.

Ethiopia, like many low- and middle-income countries, faces unique challenges in combating antimicrobial resistance (AMR) due to a combination of policy gaps, inadequate sanitation, and limited detection healthcare infrastructure. The lack of a robust national AMR surveillance system hampers timely detection and monitoring of resistance patterns, which is essential for guiding treatment protocols and policymaking (10). Furthermore, widespread antibiotic misuse in both human and veterinary medicine, driven by over-the-counter availability and poor regulation, exacerbates the issue (7). Sanitation challenges, including limited access to clean water and improper waste disposal, contribute to the spread of resistant pathogens, particularly in rural areas where healthcare access is minimal (11). These factors collectively create an environment conducive to the emergence and dissemination of AMR, highlighting the need for targeted interventions in Ethiopia that address policy enforcement, sanitation improvements, and public education on antibiotic use. Close interactions between humans, animals, and the environment, coupled with poor sanitation and limited healthcare access, create an ideal environment for the emergence and spread of resistant strains (12). A study conducted in Addis Ababa revealed that 27 out of 59 *Salmonella* isolates from stool samples exhibited resistance to three or more antimicrobials, with 17 showing resistance to five or more drugs (13). Another study on poultry in Ethiopia found that 86% of *Salmonella* isolates were multidrug-resistant, indicating that poultry serves as a significant reservoir for resistant strains (12). Environmental sources also play a crucial role in the dissemination of resistant *Salmonella*. For example, a study analyzing water and wastewater samples from urban and peri-urban areas in Ethiopia found that 85.7% of *Salmonella* isolates were resistant to ampicillin and 88.5% exhibited multidrug resistance (14, 15).

Contributing factors to antibiotic resistance in Ethiopia are multifaceted, with significant contributions from agricultural practices, environmental factors, healthcare gaps, and regulatory issues. A major contributor is the widespread use of antibiotics in livestock, where they are employed for disease prevention and growth promotion. This practice has led to the emergence of resistant strains, particularly in *Salmonella*. A systematic review also reported alarmingly high resistance rates in livestock-associated *Salmonella* isolates, including tetracycline (89.5%) and streptomycin (77.2%) (16). Furthermore, environmental contamination exacerbates the spread of AMR, with poor waste management, and water related contamination playing pivotal roles, as highlighted by studies demonstrating that contaminated water sources facilitating the transmission of resistant *Salmonella* serotypes, particularly in rural communities where access to clean water is limited (11). In addition to environmental factors, healthcare-related gaps significantly contribute to AMR. Limited diagnostic capacity and the absence of strong routine antimicrobial resistance surveillance hinder the timely detection of resistant strains, enabling their unchecked spread. As noted in the study, the lack of stringent regulations regarding antibiotic use in both human and veterinary medicine has led to widespread misuse, further accelerating the development of resistance (10).

Antibiotic-resistant *Salmonella* presents a growing public health threat in Ethiopia, affecting humans and animals. Delays in diagnosis and limited treatment options worsen health outcomes and put additional pressure on healthcare systems. This study aims to assess the AMR profiles of *Salmonella* isolates from clinical, food, and environmental sources in Addis Ababa and surrounding areas, with a particular focus on multidrug-resistant strains. The objective is to provide a comprehensive and updated analysis of AMR in *Salmonella* by utilizing current Clinical Laboratory Standard Institute (CLSI) criteria, filling a gap in prior studies that were limited to a range of sample sources. By examining *Salmonella* isolates from multiple settings, the study seeks to contribute to a better understanding of the distribution and patterns of multidrug resistance in *Salmonella* in Ethiopia. The findings of this study will inform public health strategies, enhance surveillance efforts, and support antimicrobial resistance control, particularly in resource-limited environments.

## MATERIALS AND METHODS

### Sampling sites

The study sample was collected from Addis Ababa and neighboring towns, including Bishoftu, Dukem, Modjo, Burayu, and Holeta, areas significantly impacted by drug-resistant pathogens. Addis Ababa faces challenges from the overuse and misuse of antibiotics in both healthcare and agriculture, which contribute to the development of antimicrobial resistance. The surrounding towns are particularly vulnerable due to uncontrolled antibiotic use in home poultry farms, cattle fattening, and agricultural practices. In addition to these practices, inadequate waste management, industrial activities, and the presence of slaughterhouses further facilitate the spread of drug-resistant *Salmonella* strains in both food and environmental sources. This study aims to assess the extent and distribution of antimicrobial resistance in *Salmonella* across these regions, providing a comprehensive analysis of how human, animal, and environmental factors contribute to the emergence of multidrug-resistant pathogens.

### Sample source and isolation

A cross-sectional study design was employed to investigate the presence of *Salmonella* spp. across clinical, food, and environmental sources in Addis Ababa and surrounding towns. Stool (clinical), meat (lymph node and intestine from cattle and poultry), and environmental samples (wastewater, conveyor, cutting board, and knife swab) were systematically collected from Addis Ababa city and surrounding towns using a systematic sampling technique. The sampling was conducted across 12 health facilities and two cattle slaughterhouses. The sample size was determined based on a previously reported combined prevalence of 10% for *Salmonella* across clinical, food, and environmental sources. Using this estimate, a total of 478 samples were initially calculated; however, to account for potential non-responses and to improve representativeness, the final number of samples collected was increased to 552. A total of 552 samples were collected, including 386 stool samples from patients presenting with clinical diarrhea and 166 samples from food (meat) and environmental sources. The food and environmental samples were obtained from slaughterhouse wastewater, conveyor swabs, knife swabs, cutting surface swabs, and chicken droppings from markets. Systematic random sampling was applied at slaughterhouses and markets, with samples collected every 5 days to ensure a representative and unbiased selection. Samples were collected using sterile swabs and containers, with approximately 1 mL or 1 g collected. All specimens were placed in appropriate transport media, maintained under cold chain conditions, and processed within 2–4 hours of arrival in main laboratory. Approximately one loopful of each sample was used for culture on selective media. Prior to data collection, permissions for food and environmental sampling were obtained from the relevant authorities.

To enhance the recovery of *Salmonella*, samples were first enriched using selenite broth, following enrichment, streaked onto MacConkey and xylose lysine deoxycholate (XLD) agar for selective isolation and differentiation. Presumptive *Salmonella* colonies were identified based on their characteristic morphology on XLD agar, which includes red colonies with or without black centers. Further confirmation of *Salmonella* was carried out using a series of biochemical tests to ensure accurate identification. The triple sugar iron agar test was used to assess the isolates’ ability to ferment carbohydrates and produce hydrogen sulfide, followed by the urease test to determine the presence of urease enzyme activity. The citrate utilization test was used to evaluate the capability of the isolates to utilize citrate as a sole carbon source. Additionally, the lysine iron agar test was employed to examine lysine decarboxylation and hydrogen sulfide production, providing further confirmation of the isolates’ identity as *Salmonella*. Furthermore, isolates were identified for *Salmonella* specificity based on agglutination using Mast Assure polyvalent *Salmonella* antisera (Mast Group Ltd., UK), which target the O-antigen of *Salmonella*. This screening process ensures high specificity in identifying *Salmonella* isolates from other bacterial species.

### Antibiotic susceptibility testing

The confirmed *Salmonella* isolates were subjected to antibiotic susceptibility testing using standardized protocols. Initially, isolates were subcultured on XLD agar overnight, then transferred to tryptic soy agar and incubated for 24 hours at 35°C. After incubation, bacterial suspensions were prepared in sterile 0.85% normal saline to achieve a turbidity equivalent to a 0.5 McFarland standard.

Sterile cotton swabs were used to spread the bacterial suspensions evenly over the surface of Mueller-Hinton agar plates (High Media, UK) prepared in 120 mm Petri dishes. The Kirby-Bauer disk diffusion method was employed to study the susceptibility of the isolates to 10 commonly used antibiotics: ampicillin (10 µg), ciprofloxacin (5 µg), ceftriaxone (30 µg), ceftazidime (30 µg), amoxicillin-clavulanic acid (AMC) (20/10 µg), tetracycline (30 µg), gentamicin (GEN) (10 µg), trimethoprim-sulfamethoxazole (SXT) (1.25/23.75 µg), azithromycin (15 µg), and chloramphenicol (30 µg) (Oxoid, UK). The inoculated plates were left at room temperature to dry for 3 to 5 minutes and the antibiotic disks were aseptically placed on the inoculated agar plates using sterilized forceps, ensuring a minimum distance of 25 mm from each other (center to center). The plates were incubated at 37°C for 24 hours, after which the zones of inhibition around each antibiotic disk were measured using a ruler. As a control, standard *Salmonella Typhimurium* strain ATCC 14028 was included in the test analysis for each drug used. Antibiotic susceptibility results were interpreted using CLSI guidelines 2024 version (17). The zone diameters were classified as susceptible, intermediate, or resistant based on the antibiotic-specific breakpoints. This methodology ensured reliable and reproducible results, providing data on the resistance profiles of the *Salmonella* isolates.

### Data analysis

The collected data underwent initial cleaning procedures, including the identification and correction of missing values, removal of duplicate entries, and verification of data consistency to ensure accuracy. Following data cleaning, the data set was entered into SPSS version 25 for statistical analysis. Descriptive statistics, including frequencies and percentages, were calculated to summarize the distribution and characteristics of the study variables. Comparative analysis of antimicrobial resistance patterns was performed using the χ^2^ test, with statistical significance reported where applicable.

## RESULTS

The results presented below offer a detailed breakdown of the frequency and percentage of positive *Salmonella* samples identified from each sampling site. This analysis helps to pinpoint high-risk areas where contamination is more prevalent, thereby informing food safety policies, public health strategies, and hygiene interventions.

**Fig 1:**
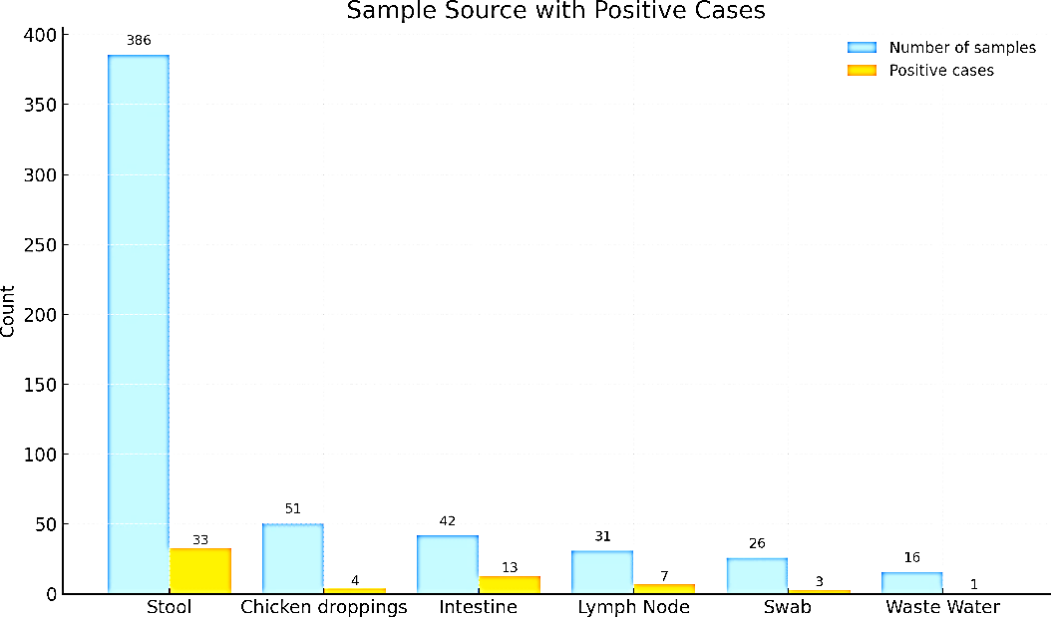
Distribution of sample type with their respective positivity rate. The bar chart shows the number of samples collected from various sources (blue bars) and the number of *Salmonella*-positive samples (orange bars). Stool samples representing clinical specimens from humans, chicken droppings obtained from marketplaces, meat samples (lymph node and intestine), and swab samples were collected from slaughterhouse environments, including conveyor belts, knives, and cutting boards. Wastewater samples were collected from slaughterhouses.

A total of 552 samples were collected from diverse sources including stool (*n* = 386), chicken droppings (*n* = 51), intestines (*n* = 42), lymph nodes (*n* = 31), swabs from surfaces (*n* = 26), and wastewater (*n* = 16) (Fig. 1). Among these, stool samples from patients with diarrhea represented the largest proportion (69.9%) of all collected samples, with a positivity rate of 8.6%. Intestinal samples, despite constituting only 8.1% of total samples, showed the highest positivity rate at 24.4%, followed by lymph node samples with a positivity rate of 21.9%. Samples from swabs, chicken droppings, and wastewater had relatively lower positivity rates of 7.4%, 7.7%, and 6.3%, respectively.

**Fig 2:**
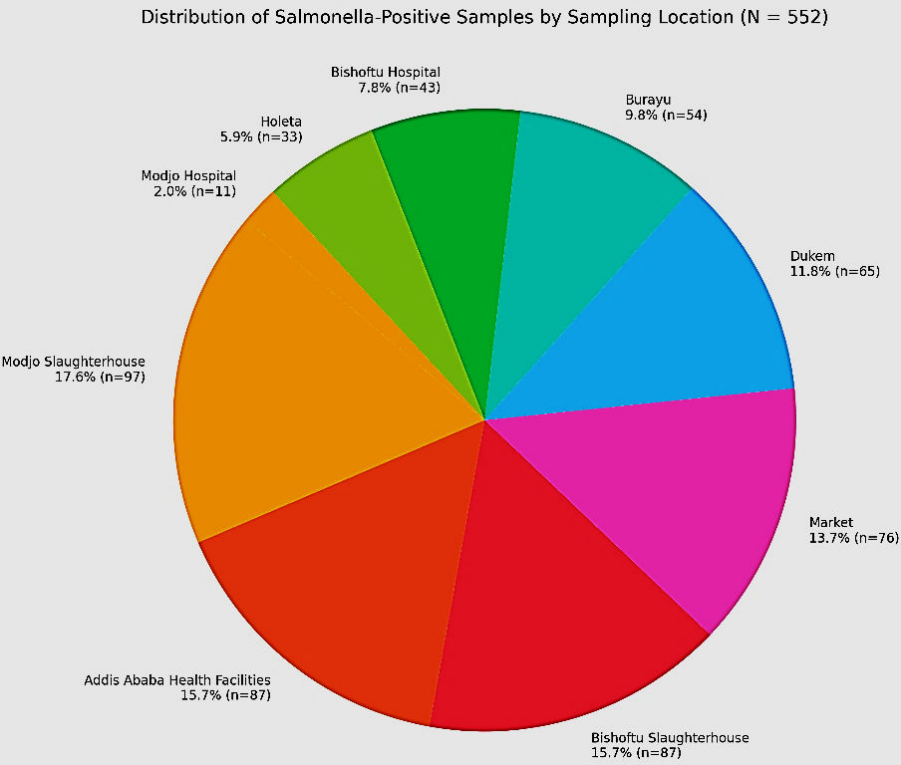
Distribution of positive samples by sampling area across a total of 552 total samples. The pie chart illustrates the proportion of *Salmonella*-positive isolates obtained from different sampling sites. Percentage represents positive cases from each sampling site, and *n* represents total samples from each location.

The figure presents the distribution of *Salmonella*-positive samples across various sampling locations from a total of 552 collected samples (Fig. 2). The highest proportion of positive cases was identified at Modjo Slaughterhouse, accounting for 17.6% (*n* = 97). Addis Ababa health facilities and Bishoftu Slaughterhouse each contributed 15.7% of positives, corresponding to 87 isolates each. Market samples accounted for 13.7% (*n* = 76), while Dukem contributed 11.8% (*n* = 65). Burayu accounted for 9.8% (*n* = 54), followed by Bishoftu Hospital with 7.8% (*n* = 43). Holeta contributed 5.9% (*n* = 33), and Modjo Hospital had the lowest share at 2.0% (*n* = 11).

Among the 61 *Salmonella* isolates tested, tetracycline showed the highest resistance rate at 63.9%, with 15.3% intermediate and only 20.8% sensitive isolates, followed by ampicillin with 50.8% resistance, 19.7% intermediate, and 29.5% sensitivity (Table 1). Azithromycin also exhibited moderate resistance at 27.9%, with 34.4% intermediate and 41.0% sensitive isolates. Trimethoprim-sulfamethoxazole and chloramphenicol showed resistance in 21.3% and 16.4% of isolates, respectively, with moderate intermediate and sensitivity levels (SXT: 14.8% intermediate, 63.9% sensitive, CHL: 23.9% intermediate, 59.7% sensitive). Ciprofloxacin resistance was comparatively low at 8.2%, though 37.7% of isolates showed intermediate susceptibility and 54.1% were sensitive. Among beta-lactams, ceftriaxone and amoxicillin-clavulanic acid exhibited low resistance (6.6% and 8.2%, respectively), with high sensitivity rates (78.6% and 80.3%), although only ceftriaxone showed a statistically significant distribution for AMC. Ceftazidime had 8.2% resistance, 31.1% intermediate, and 60.7% sensitivity. Gentamicin demonstrated the lowest resistance rate at 3.3%, with 1.6% intermediate and 95.1% sensitivity, and no significant variation across samples. Statistically significant differences in resistance patterns were observed for most antibiotics tested (*P <* 0.05), particularly for tetracycline, ampicillin, azithromycin, trimethoprim-sulfamethoxazole, chloramphenicol, ceftriaxone, ciprofloxacin, and ceftazidime, indicating non-random distribution across isolates.

**TABLE 1:** Susceptibility to drug classes with significance value based on CLSI breakpoints^*a*^

Drug class	Antibiotics	Resistant, *N* (%)	Intermediate, *N* (%)	Sensitive, *N* (%)	*P*-value
Fluoroquinolones	Ciprofloxacin	8.2	37.7	54.1	*0.0495*
Beta-lactams	Ampicillin	50.8	19.7	29.5	*<0.0001*
	Ceftriaxone	6.6	14.8	78.6	*0.0051*
	Amoxicillin- clavulanic acid	8.2	11.5	80.3	*0.0651*
Macrolides	Azithromycin	27.9	31.1	41.0	*<0.0001*
Tetracyclines	Tetracycline	63.9	15.3	20.8	*<0.0001*
Aminoglycosides	Gentamicin	3.3	1.6	95.1	*0.8721*
Phenicols	Chloramphenicol	16.4	23.9	59.7	*0.0038*
Sulfonamides	Trimethoprime- sulfamethoxazole	21.3	14.8	63.9	*<0.0001*
Cephalosporins	Ceftazidime	8.2	31.1	60.7	*0.0051*

^*a*^ Note: *P*-values were calculated using χ^2^ to assess the statistical significance of resistance pattern distributions across each antibiotic and antibiotic class.

In this study, multidrug resistance was defined as resistance to three or more classes of antimicrobial agents, following internationally accepted criteria. Multidrug-resistant isolates were predominantly recovered from human stool samples of patients with diarrhea, with additional isolates detected in poultry and slaughterhouse samples, including meat (Table 2). Resistance spanned a wide range of antibiotic classes: fluoroquinolones, sulfonamides, beta-lactams, tetracyclines, phenicols, macrolides, and aminoglycosides, with some isolates exhibiting resistance to as many as seven antibiotics, including CIP, sulfonamides (SXT), AMP, TET, CHL, AMC, and GEN. Clinical isolates tended to show broader resistance profiles, including resistance to critically important drugs such as cephalosporins (ceftriaxone) and aminoglycosides, whereas isolates from poultry and slaughterhouse environments more frequently showed resistance to macrolides; azithromycin (AZM), tetracyclines, and beta-lactams. Notably, six MDR isolates originated from non-human sources. No statistically significant association was observed between sample source and resistance pattern (*P* > 0.05).

**TABLE 2:** Multidrug resistance frequency specific to drug types

Sample source	Number of resistant drugs	Class of drugs
Human	6	Fluoroquinolones, sulfonamides, beta-lactams, tetracyclines, phenicols, beta-lactams
	7	Fluoroquinolones, sulfonamides, beta-lactams, tetracyclines, phenicols, beta-lactams, aminoglycosides
	5	Fluoroquinolones, sulfonamides, tetracyclines, phenicols, beta-lactams
	7	Cephalosporins, sulfonamides, beta-lactams, tetracyclines, phenicols, beta-lactams, aminoglycosides
	5	Cephalosporins, sulfonamides, tetracyclines, phenicols, aminoglycosides
	5	Macrolides, sulfonamides, beta-lactams, tetracyclines, phenicols
	4	Sulfonamides, beta-lactams, tetracyclines, beta-lactams
	3	Beta-lactams, tetracyclines, beta-lactams
Poultry (intestine/droppings)	5	Fluoroquinolones, macrolides, sulfonamides, tetracyclines, phenicols
	4	Fluoroquinolones, macrolides, sulfonamides, phenicols
	3	Macrolides, beta-lactams, tetracyclines
Lymph node	6	Cephalosporins, sulfonamides, beta-lactams, tetracyclines, phenicols, beta-lactams
	3	Macrolides, beta-lactams, tetracyclines

Among the 61 *Salmonella* isolates tested, tetracycline exhibited the highest resistance rate, with 39 isolates (63.9%) resistant (Table 3). Resistance was most pronounced in stool samples (22/33; 66.7%) and poultry intestine samples (6/13; 46.2%). Ampicillin followed closely, with 34 resistant isolates (55.7%), mainly from stool (19/33; 57.6%) and lymph node (6/7; 85.7%) sources. Moderate resistance was recorded for azithromycin (17/61; 27.9%), with the highest proportions from stool (8/33; 24.2%), poultry intestine (4/13; 30.8%), and chicken droppings (3/4; 75.0%). Trimethoprim-sulfamethoxazole and chloramphenicol resistance were found in 15 (24.6%) and 12 (19.7%) isolates, respectively, both frequently identified in stool and swab samples. Lower resistance was observed for amoxicillin-clavulanic acid (6/61; 9.8%), mostly from stool, poultry, and droppings. Ceftriaxone and ciprofloxacin resistance were 4 (6.6%) and 5 (8.2%), respectively, while gentamicin showed the lowest resistance at two isolates (3.3%), with minimal distribution across sources. Chi-square analysis revealed statistically significant differences in resistance across sample types for tetracycline (*P* = 0.023) and ampicillin (*P* = 0.029). Other antimicrobials showed no statistically significant variation, although some, such as azithromycin, indicated non-significant trends that warrant further monitoring.

**TABLE 3:** Distribution of antimicrobial-resistant *Salmonella* isolates by sample type and drug type based on CLSI standards^*a*^

Sample type	AMC n(%)	CHL n(%)	TET n(%)	AMP n(%)	CIP n(%)	AZM n(%)	SXT n(%)	CRO n(%)	GEN n(%)
Swab	1(33.3)	2(66.7)	2(66.7)	2(66.7)	0(0)	2(66.7)	1(33.3)	1(33.3)	0(0)
Poultry intestine	1(7.7)	2(15.4)	6(46.2)	4(30.8)	2(15.4)	4(30.8)	2(15.4)	0(0)	0(0)
Chicken droppings	1(25.0)	2(50.0)	4(100)	4(100)	0(0)	3(75.0)	2(50.0)	0(0)	0(0)
Lymph node	0(0)	0(0)	4(57.1)	6(85.7)	0(0)	1(14.3)	1(14.3)	1(14.3)	0(0)
Stool	3(9.1)	6(18.2)	22(66.7)	19(57.6)	3(9.1)	8(24.2)	9(27.3)	2(6.1)	2(6.1)
Wastewater	0(0)	0(0)	1(100)	0(0)	0(0)	0(0)	0(0)	0(0)	0(0)
Total n(%)	6(9.8)	12(19.7)	39(63.9)	34(55.7)	5(8.2)	17(27.9)	15(24.6)	4(6.6)	2(3.3)
*P-value*	*0.976*	*0.063*	*0.023*	*0.029*	*0.999*	*0.256*	*0.502*	*0.865*	*0.423*

^*a*^ Note: ceftriaxone (CRO), chloramphenicol (CHL), tetracycline (TET), ampicillin (AMP), ciprofloxacin (CIP), gentamicin (GEN), Trimethoprime-sulfamethoxazole (SXT), Azithromycin (AZM).

## DISCUSSION

The study highlights a significant public health concern regarding antimicrobial resistance in *Salmonella* isolates from human, food, and environmental sources in Ethiopia. The findings demonstrate varying levels of resistance across a range of commonly used antibiotics. Notably, high resistance rates were observed for tetracycline (63.9%) and ampicillin (55.7%), with statistically significant differences in resistance patterns across sample types (*P =* 0.023 and *P =* 0.029, respectively). The notably high resistance rates to tetracycline and ampicillin, particularly among clinical isolates, highlight a critical challenge for empirical treatment of *Salmonella* infections in Ethiopia, as these antibiotics are widely used as first-line therapies in many low-resource settings. The significant variation in resistance patterns across sample types further underscores the need for source-specific surveillance and updated treatment guidelines that reflect local resistance trends.

Moderate resistance was also evident for trimethoprim-sulfamethoxazole, while ciprofloxacin resistance remained comparatively low despite a substantial proportion of isolates showing intermediate susceptibility. Encouragingly, ceftriaxone, gentamicin, and amoxicillin-clavulanic acid exhibited low resistance rates and retained high levels of efficacy against the isolates tested. These significant variations in resistance patterns indicate genuine differences in susceptibility profiles rather than random distribution, emphasizing the need for ongoing antimicrobial resistance surveillance and prudent antibiotic use. These findings align with recent global studies in India, which found that fluoroquinolone resistance in *Salmonella* has been steadily rising, posing challenges in treatment ( 18). The global context of resistance to fluoroquinolones is concerning, as these are first-line treatment options for *Salmonella* infections. A study from Brazil in 2024 revealed that *Salmonella* resistance to ciprofloxacin increased significantly from 12% in 2020 to 22% in 2024 (4), highlighting an alarming global trend that mirrors the findings in this current study. This increase in fluoroquinolone resistance is often linked to the overuse and misuse of antibiotics in both human health and agriculture, as pointed out in a 2023 study from China (19), where agricultural antibiotic use has exacerbated the emergence of resistant strains.

Study findings in South Africa indicated similar trends where *Salmonella* strains resistant to tetracycline were prevalent in both human and animal samples (20). The study emphasized the need for stronger surveillance and regulation in agricultural practices to curb the rise in drug resistance. In Ethiopia, the high resistance to tetracycline suggests a possible over-reliance on this antibiotic, especially in veterinary and agricultural settings, which could be contributing more to the problem (8, 21). Thus, tetracycline’s frequent use in livestock necessitates regulatory action to limit non-therapeutic applications. In contrast, gentamicin showed a very high sensitivity rate of 95.1% in this study, consistent with recent reports from the USA and Europe where gentamicin remains highly effective against *Salmonella* (22, 23). However, the emergence of multidrug-resistant strains, as evidenced by the data on resistance to up to seven antibiotics in certain isolates, demonstrates the urgent need for alternative treatments. A recent study in the UK (24) also highlighted the effectiveness of gentamicin but stressed the rising concern of resistance developing even to aminoglycosides, including gentamicin, due to overuse in both human and animal health sectors. From this study finding, gentamicin’s high sensitivity rate underscores its value as a frontline therapy; however, cautious use is essential to prevent emerging resistance.

The study from Ethiopia also found that environmental and food samples contributed significantly to the spread of resistance, with 6 out of 18 multidrug-resistant isolates originating from these sources. Studies have reported varying levels of antimicrobial resistance in *Salmonella* isolates from different sources (25, 26). A systematic review conducted in Ethiopia highlighted significant resistance to commonly used antibiotics, including ampicillin, tetracycline, and chloramphenicol, with multidrug-resistant strains being prevalent (27). In a study conducted in central Ethiopia, *Salmonella* isolates from poultry farms exhibited resistance to multiple antibiotics, including ampicillin, tetracycline, and sulfamethoxazole-trimethoprim (28). Another study focusing on NTS in Addis Ababa reported resistance to ampicillin, chloramphenicol, and tetracycline among isolates from patients with diarrhea (29).

This finding aligns with the results of a similar study in Kenya, which found that antimicrobial resistance in *Salmonella* was not confined to clinical settings but was widespread in the food supply chain, contributing to the transmission of resistant strains to humans (30). High AMR rates in Ethiopia are alarming, similar to studies from neighboring countries reflecting similar challenges. The One Health approach, which considers the interconnectedness of human, animal, and environmental health, is critical in addressing this issue, which highlighted the importance of coordinated efforts across sectors to reduce the burden of antimicrobial resistance globally (31, 32). The finding reinforces the global challenge of antimicrobial resistance in *Salmonella*, particularly concerning high resistance rates to tetracycline and ampicillin. These results, coupled with recent international data, underscore the need for continuous surveillance, better antimicrobial stewardship, and a One Health approach to combat multidrug-resistant pathogens. Given the widespread nature of resistance, both in clinical and environmental settings, the development of new antibiotics and alternative treatment strategies must be prioritized to mitigate the growing threat posed by antimicrobial resistance. The multidrug-resistant isolates in Table 2 were predominantly recovered from human stool samples, while additional MDR isolates from poultry and meat suggest potential cross-transmission, emphasizing the need for robust One Health surveillance. On the other hand, the detection of multidrug resistance in a substantial proportion of isolates, alongside increasing resistance to azithromycin (27.9%), a key alternative treatment, suggests an evolving resistance landscape. The presence of azithromycin resistance in food-related sources (poultry and chicken droppings) implies potential zoonotic and foodborne transmission of resistant strains. These findings call for enhanced AMR monitoring, stricter antibiotic stewardship, and integrated One Health approaches to mitigate further spread.

The distribution of antimicrobial resistance among isolates reveals notable associations between specific drug types and isolate sources. Accordingly, ampicillin and tetracycline resistance were strongly associated with stool and meat sources, with the highest resistant isolate counts from stool and meat. This suggests frequent exposure to these drugs in clinical and food production settings. Chloramphenicol and trimethoprim-sulfamethoxazole resistance were also common in stool samples, while ciprofloxacin showed low resistance across all sources. Azithromycin and ceftriaxone resistance were less frequent, mainly in stool and meat. Significant associations were found for ampicillin (*P* = 0.004) and tetracycline (*P* = 0.036), indicating source-specific resistance patterns. These findings are consistent with studies that identified food animals and wastewater as critical reservoirs for multidrug-resistant *Salmonella* in Ethiopia and other low-resource settings (12, 33).

By integrating routine surveillance and infection control programs, timely intervention strategies could be implemented, reducing the burden of MDR *Salmonella* and improving patient outcomes. We acknowledge that the study is limited to phenotypic resistance profiling using disk diffusion, and molecular characterization of resistance genes was not performed. In addition, multiple antibiotic resistance comparisons were made without statistical adjustment, which may increase the risk of type I error. Therefore, future studies should focus on the development of rapid point-of-care diagnostic kits for ease of detection, molecular characterization of resistance genes, longitudinal surveillance of antimicrobial resistance trends, and assessment of antimicrobial stewardship program effectiveness. These efforts will enhance case detection, improve understanding of resistance mechanisms, and support the design of more targeted and effective control strategies.

### Conclusion

This study highlights the critical issue of antimicrobial resistance in *Salmonella* isolates from Ethiopia, revealing significant resistance to tetracyclines, and ampicillin, which poses a growing threat to effective treatment regimens both locally and globally. While antibiotics such as gentamicin and ceftriaxone remain effective, the rise of multidrug-resistant strains underscores the urgent need to enhance AMR surveillance, promote rational antibiotic use, and adopt a One Health approach to address resistance across human, animal, and environmental health sectors. The widespread resistance, particularly from food and environmental sources, calls for strengthened antimicrobial stewardship, stricter surveillance, and exploration of alternative therapies.

In conclusion, addressing AMR requires immediate actions to curb antibiotic misuse, strengthen infection control measures, and prioritize public health initiatives. The detection of multidrug-resistant *Salmonella* across human, poultry, and environmental sources highlights the need for enhanced surveillance systems to monitor resistance patterns and inform targeted intervention strategies. Urgent implementation of national-level antibiotic stewardship programs is essential to support these efforts. However, the findings are limited to the specific study setting (urban and semi-centers) and the cross-sectional data collected during this period, which may not fully represent broader or long-term trends. Future research should focus on simple and effective point-of-care diagnostics development, molecular characterization of resistance genes, longitudinal monitoring of AMR trends, and evaluating the impact of intervention strategies, such as antibiotic stewardship programs, to strengthen control efforts.

### Study limitation

This study provides valuable insights into the current status of AMR in *Salmonella* in Ethiopia, but its findings are limited to urban areas, which may not fully represent national antimicrobial susceptibility patterns. Additionally, the cross-sectional design limits the ability to analyze trends over time, and the study did not investigate the molecular mechanisms underlying resistance.

## Data Availability

The meta data sets used during this study available at https://zenodo.org/records/15839222.
